# Assessing the Psychological Impacts of COVID-19 in Undergraduate Medical Students

**DOI:** 10.3390/ijerph18062952

**Published:** 2021-03-13

**Authors:** Alyssa A. Guo, Marissa A. Crum, Lauren A. Fowler

**Affiliations:** Department of Biomedical Sciences, School of Medicine Greenville, University of South Carolina, Greenville, SC 29605, USA; aaguo@email.sc.edu (A.A.G.); mcrum@email.sc.edu (M.A.C.)

**Keywords:** COVID-19, undergraduate medical education, stress, anxiety, psychological impacts, mental health

## Abstract

Medical education has been uniquely affected by the Novel Coronavirus Disease 2019 (COVID-19). As the pandemic’s psychological impacts on medical students remain unclear, this study assessed COVID-19’s impacts on undergraduate medical students’ stress and anxiety. A nationwide, online survey was administered via email chains between June-August 2020 to first-fourth year medical students in the United States. Demographics, 4-point Perceived Stress Scale that measures stress, 7-point Generalized Anxiety Disorder Scale that measures anxiety, and the impacts of social, health, and academic stressors due to COVID-19 were collected. Of the 852 students who participated, 66.1% experienced mild, moderate, or severe anxiety. Mean PSS-4 score was 7.25/16. Stress was highest in second- through fourth-year students. Students with preexisting mental health conditions had significantly higher stress and anxiety scores, and higher percentage of stress attributed to COVID-19. Trust in government institutions during COVID-19 was the highest stressor in first- and second-year students. Delay/availability of standardized exams was the highest stressor for third-year students. Impact on rotations/residencies was the highest stressor for fourth-year students. Understanding how students’ anxiety and stress have changed due to COVID-19 will allow educators to identify students in need and guide recommendations on the implementation of psychological interventions and support strategies.

## 1. Introduction

The Novel Coronavirus Disease 2019 (COVID-19), declared a pandemic by the World Health Organization in March 2020, spread quickly worldwide and impacted all aspects of daily life. U.S. undergraduate medical students were uniquely affected by the COVID-19 pandemic due to their positions both as students and future physicians. Undergraduate medical students refer to students in the professional degree programs, Medical Doctorate (M.D.) and Doctor of Osteopathic Medicine (D.O.), who are currently in training to become physicians. Due to the need for social distancing, learning opportunities at all levels of training were disrupted and adapted [1,2]. The pandemic’s social and economic consequences were severe, and as a result, collective mental health suffered [3]. Prior studies show that life disruptions as a result of natural disasters and prior epidemics lead to significant mental illness increases [4]. With the unprecedented nature of the pandemic impacting students at all levels of training, immediate data collection is needed to identify the psychological impacts of the COVID-19 pandemic in undergraduate medical students. 

Numerous studies document the high prevalence of depression and depressive symptoms in medical students in the United States [5]. Dyrebye and colleagues found that prior to COVID-19, medical students, from the United States (U.S.) and Canada, experienced higher levels of emotional distress than their age-matched peers [6]. Rotenstein and colleagues found that United States medical students in the age categories of 18–25 and 26–49 have 2.2 and 5.2 times, respectively, higher chances of having depressive symptoms compared to the prevalence of depressive symptoms in 9.3% and 7.2% of the general public [7]. As we continue to experience the impact of COVID-19, a meta-analysis demonstrated that the COVID-19 pandemic had significant mental health impacts on medical students in China, United Arab Emirates, Iran, Brazil, and India [8]. To the best of our knowledge, there have not been studies addressing COVID-19’s impacts on U.S. medical students’ mental health nor any studies on identifying stressors specific to each class during the pandemic. 

One of the challenges for medical school administrations in addressing the pandemic’s psychological impact is that each class level had different curricular demands and thus sources of stress that are exacerbated by the COVID-19 pandemic. In a typical U.S. curriculum, medical students progress through two preclinical years and two clinical years of hospital clerkships. Multiple studies show that anxiety symptoms are highest in third-year medical students, the usual transition from preclinical to clinical years [5,9]. In January 20, 2020, the first cases of COVID-19 were reported in the United States [10]. To conserve personal protective equipment, the American Association of Medical Colleges released guidelines recommending suspension of clinical activities involving direct patient care [11]. By March of 2020, many schools cancelled hospital clerkships and transitioned curriculums to online modules and simulated patient care experiences, which may have added to the anxiety students were already experiencing. Students at other levels were also affected, with preclinical students learning remotely online. Closures caused testing sites to repeatedly reschedule medical licensing exams [12]. Furthermore, the process of applying for a residency position was modified to accommodate social distancing guidelines [13]. These numerous last-minute adaptations undoubtedly impacted student learning and added stress. Therefore, there is a gap in knowledge in identifying specific academic stressors each school year faces, and these stressors may include reduced quality of clinical educational experience, challenges with remote learning, delay and availability of standardized exams, and the pandemic’s impacts on rotations and residency placements.

Aside from curricular disruptions, students were also coping with new stressors related to life’s realities during a pandemic. Many experienced grief due to loss of loved ones, loneliness due to social distancing practices, anxiety over the risk of infection, and financial insecurity [14,15,16,17]. Since the beginning of the COVID-19 pandemic, the United States has faced a severe shortage of personal protective equipment and needed to ration among healthcare workers [18]. Health concerns such as illness, death and disability, and personal protective equipment availability may be contributing to increased stress while students are in the clinic. Students’ stress may also be additive with stressors from outside the clinic. Social stressors such as COVID-19’s impact on daily life, poor social distancing practices of others, and decreased trust in government institutions’ handling of COVID-19 may all contribute to heightened stress levels in medical students. Identifying unique academic, health, and social stressors that are negatively impacting students’ health is crucial for targeted solutions and interventions. 

The present study assesses COVID-19’s impacts on stress and anxiety and seeks to identify pandemic-related stressors that most impact medical students’ mental health at different levels of training. While many medical schools have transitioned students back to in-person activities and as many new students are transitioning into the clinical environment in the backdrop of active COVID-19 community spread, this knowledge can guide educators and school administrations’ plans on potential interventions and support strategies for students in need. Additionally, as many medical schools remain in a virtual-in-person hybrid instructional style for the unforeseeable future and uncertainty remains, medical school administrators should continue to monitor pandemic-related stressors that may be impacting students’ mental health. The ultimate goal of this study is to bring awareness of the specific needs of medical students in response to unprecedented changes in learning environments. With this knowledge, tracking the psychological impacts of COVID-19 will help determine the best paths forward during the ongoing pandemic and serve as a basis in preparing for future events that disrupt undergraduate medical education. 

## 2. Materials and Methods

### 2.1. Study Population and Sample 

Between June and August of 2020, 929 medical students from the United States, comprised of Medical Doctorate (M.D.) and Doctor of Osteopathic Medicine (D.O.) degree-pursuing students, participated in an anonymous online survey. There were 9, 7, 5, 2, and 1 school(s) represented from the South, Northeast, Midwest, West, and Atlantic regions, respectively. The respondents included 210 first, 286 second, 180 third, and 176 fourth year students who completed the survey. Email chains through school administration were the primary method to recruit participants from geographically diverse regions in the U.S. using cluster sampling. School administrators emailed the survey link to students. The survey was anonymous, confidential, and without identifiers. The study protocol was reviewed and approved by the Institutional Review Board at the University of South Carolina.

### 2.2. Rating Instruments 

A self-reported anonymous online survey collected data on demographic information and psychometric inventories including the Perceived Stress Scale (PSS-4) and Generalized Anxiety Disorder Scale (GAD-7) to assess levels of perceived stress and anxiety, respectively. Students reported how they felt about COVID-19 impacting their mental health and how they compare to their peers. Students also reported academic, health, and social stressors that were impacted by COVID-19.

#### 2.2.1. Demographic Characteristics 

The online survey collected demographic information including year in medical school, race, and whether the student had preexisting mental health conditions. 

#### 2.2.2. Perceived Stress Scale (PSS-4) 

The Perceived Stress Scale (PSS-4) was applied to assess how respondents feel they can cope with current stressors. The PSS-4 has been shown to be a valid indicator of stress levels and have been utilized in a number of studies [19,20]. The PSS-4 includes 4 items to assess how often the respondent experiences certain stressful situations. Respondents reported their frequency using a 5-point Likert rating scale ranging from 0 (never) to 4 (very often), with the total score ranging from 0–16. The higher the PSS-4 score, the less the respondent perceives that they can cope with current stressors. PSS-4 does not have cutoffs but is instead compared to normative data. The PSS-4 had also been reported in the literature tracking the impact of COVID-19 [21,22].

#### 2.2.3. Generalized Anxiety Disorder Scale (GAD-7) 

The respondents also reported their anxiety symptom frequency using the 7-point Generalized Anxiety Disorder (GAD-7) scale to assess anxiety levels at the time of the survey. The GAD-7 has been previously validated in the general population and in diverse clinical settings [23,24,25]. The GAD-7 is measured using a 4-point Likert rating scale ranging from 0 (not at all sure) to 3 (nearly every day), with the total score ranging from 0–21. Mild, moderate, and severe anxiety scores are defined as 5, 10, and 15, respectively. A score greater than 10 represents probable clinical generalized anxiety disorder. 

#### 2.2.4. Unique Stressors during the COVID-19 Pandemic

To identify the stressors caused by COVID-19 that students perceive most contributed to increased stress, students were asked to rate how they feel certain academic, health, and social stressors contributed to feelings of stress on a visual analog scale ranging from 0 (not stressful) to 10 (extremely stressful). Examples of the identified stressors include the impact of the transition to remote learning, fear of becoming ill with COVID-19, and the impact of COVID-19 on daily life. 

#### 2.2.5. Perceptions of Stress and Anxiety

Using a visual analog scale ranging from 1 (much less) to 5 (much more), respondents were asked to recall how stressed or anxious they felt at the time of the survey compared to a time point before major lockdowns in the United States (this is referred to as changes in anxiety levels since March 2020 where students were asked “have you noticed any changes in your anxiety levels since March 2020?”). 

They were also asked to report how stressed and anxious they feel compared to their peers [this is referred to as anxiety/stress compared to other students (before COVID) where the students were asked “how anxious or stressed do you feel compared to other medical students?” using the aforementioned visual analog scale]. 

Students were also asked what percentage of their stress they thought was caused by COVID-19 (this is referred to as percentage of stress due to COVID-19).

### 2.3. Data Analysis 

Data analysis was performed using SPSS Version 26.0 (IBM Corporation, Armonk, NY, USA). Descriptive statistics were conducted to describe demographic and other related respondent characteristics. Stress and anxiety data were not normally distributed across years in school (according to Levene’s test for equality of variances), and therefore, non-parametric analyses were used. Kruskal Wallis was used to evaluate whether demographic information, such as the year in school and preexisting mental health conditions show a significant difference in relation to stress and anxiety. Relationships between demographic characteristics and stressors were assessed using Spearman’s Rho. Linear regressions were performed to assess academic, health, and social stressors that contributed most to PSS-4 and GAD-7 across year in school. Statistical significance was defined as a two-tailed *p* < 0.05. 

## 3. Results 

### 3.1. Sample Characteristics 

A total of 929 students from 24 medical schools participated, with a completion rate of 91.7%. During the 2019-2020 academic year, there were 30,367 Doctor of Osteopathic Medicine (D.O.) and 92,674 Medical Doctorate (M.D.) degree-pursing medical students [26,27]. Therefore, the present study represents 0.755% of the total number of enrolled medical students. Students’ year in school and race are characterized in Table 1. 

### 3.2. Anxiety in Undergraduate Medical Students 

**Figure 1:**
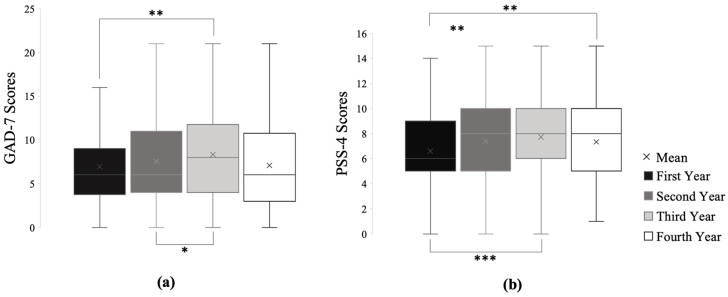
PSS-4 and GAD-7 Scores across Different School Years. (**a**) GAD-7 cutoffs for mild is 5, moderate is 10, and severe anxiety is 15. Third year students reported significantly higher GAD-7 scores than both first (*p* < 0.01) and second year students (*p* < 0.05). Mean GAD-7 scores (SD) for first-fourth year students are 6.94 (4.90), 7.54 (5.33), 8.33 (5.56), and 7.09 (5.20), respectively. The overall GAD-7 score (SD) for all respondents is 7.47 (5.27). (**b**) First year students reported significantly lower PSS-4 scores than second (*p* < 0.01), third (*p* < 0.001), and fourth year (*p* < 0.01) students. Mean (standard deviation/SD) PSS-4 scores for first-fourth year students are 6.60 (2.59), 7.39 (3.23), 7.72 (3.20), and 7.33 (2.98), respectively. The overall PSS-4 score (SD) for all respondents is 7.25 (3.05). * *p* < 0.05, ** *p* < 0.01, *** *p* < 0.001.

In first through fourth year medical students, 289 (33.92%) did not have any symptoms of anxiety (based on GAD-7), and 298 (34.98%) showed mild anxiety, 164 (19.25%) had moderate anxiety, and 101(11.85%) had severe anxiety. Table 2 details anxiety levels reported in each class. Figure 1a indicates anxiety scores in each class and the overall anxiety score. Figure 1a also indicates that third year students reported significantly higher anxiety levels than both first year (*p* < 0.01) and second year students (*p* < 0.05).

Self-reported changes in anxiety levels since March 2020 was significantly different for students of different years in school (*p* < 0.01). Specifically, changes in anxiety levels as compared between first and third years, and between second and third years, were significant (*p* < 0.015, *p* < 0.013, respectively). 

### 3.3. Stress in Undergraduate Medical Students 

Figure 1b shows the stress levels across different classes as measured by PSS-4. Overall, the mean PSS-4 score was 7.25/16 (SD = 3.05). First year students had statistically lower levels of stress than that of second, third, and fourth year students (*p* < 0.05). 

Furthermore, the results also delineated academic, social, and health stressors unique to each class. Trust in government institutions and others’ social distancing practices were the highest rated stressors in first years (mean ± SD, 6.67 ± 2.64, 6.30 ± 2.53, respectively) and second years (6.99 ± 2.68, 6.52 ± 2.56, respectively). Delay and availability of standardized exams was the highest stressor for third years (7.38 ± 3.24) and the second highest stressor for fourth years (7.22 ± 2.93). Impact on rotations and residencies was the highest stressor for fourth years (7.75 ± 2.89). Table 3 delineates the measured stressors across different classes. 

### 3.4. Predictors of Stress and Anxiety 

Correlations and linear regressions were conducted to assess factors that contributed the most to GAD-7. Students who reported higher changes in anxiety levels since March 2020 were found to have higher GAD-7 scores, rho (852) = 0.572, *p* < 0.001. Students who rated high levels of stress due to the impacts of COVID-19 on daily life were also likely to have higher GAD-7 scores, rho (852) = 0.532, *p* < 0.001. 

Students who reported higher changes in anxiety levels since March 2020 had higher PSS-4 scores, rho (852) = 0.492, *p* < 0.001. Students who rated higher levels of stress due to impacts on daily life had higher PSS-4 scores, rho (852) = .496, *p* <0.001. Additionally, a linear regression was calculated to predict perceived stress based on year in school. A significant regression equation was found, F (19, 625) = 34.41, *p* < 0.001, with an R^2^ of 0.511. The students’ year in medical school was found to be a strong predictor of stress levels at the time of the survey, with third year students experiencing higher levels of stress than other years. Additionally, students who rated high levels of stress due to the remote learning transition had higher PSS-4 scores (*p* > 0.05). 

A one-way MANOVA assessed year in school’s effect on the students’ rating of percentage of stress and anxiety that is caused by COVID-19. Results show that there were no significant effect, F (1,3) = 1.58, *p* > 0.05. Though, first year medical students had the lowest rated percentage of stress and anxiety that is caused by COVID-19.

### 3.5. Effects of Preexisting Mental Health on Stress and Anxiety

Students who self-reported pre-existing mental health conditions had higher PSS-4 (*p* < 0.001) and GAD-7 scores (*p* < 0.001) (Table 4). Furthermore, preexisting mental health conditions contributed to higher percentage of stress due to COVID-19 (*p* < 0.05).

### 3.6. Perceptions of Stress and Anxiety 

Table 5 details the effects of perceptions of stress and anxiety. 

Self-reported anxiety compared to other students before COVID-19 is directly related to self-reported stress compared to other students before COVID-19, changes in anxiety levels since March 2020, changes in stress levels since March 2020, PSS-4 scores, and GAD-7 scores [rho (852) = 0.715, 0.405, 0.327, 0.492, 0.572, respectively. All *p*-values are < 0.01]. 

Self-reported stress compared to other students before COVID-19 is directly related to changes in anxiety levels since March 2020, changes in stress levels since March 2020, PSS-4 scores, and GAD-7 scores [rho (852) = 0.351, 0.415, 0.496, 0.532, respectively. All *p*-values are < 0.01]. 

Change in anxiety levels since March 2020 is directly related to self-reported anxiety and stress compared to other students before COVID-19, change in stress levels since March 2020, PSS-4 scores, and GAD-7 scores [rho (852) = 0.405, 0.351, 0.751, 0.551, 0.570, respectively. All *p*-values are < 0.01]. 

Change in stress levels since March 2020 is directly related to self-reported anxiety and stress compared to other students before COVID-19, change in anxiety levels since March 2020, PSS-4 scores, and GAD-7 scores [rho (852) = 0.327, 0.415, 0.751, 0.553, 0.516, respectively. All *p*-values are < 0.01].

## 4. Discussion

In April 2020, a group of 24 world-leading experts from across the bio-psycho-social spectrum of expertise, determined that there is an immediate priority in collecting data on the mental health effects of the COVID-19 pandemic across different populations [28]. The present study answers the call by determining the impact of COVID-19 in the undergraduate medical student population in the U.S. This study reports that 66.1% of undergraduate medical students in the U.S., across 24 institutions, are experiencing mild to severe anxiety. As expected, prevalence of anxiety is higher than the previously reported anxiety levels. A prior meta-analysis estimated the global prevalence of anxiety in medical students at 33.8% [29]. A prior study, of medical students in the U.S., found anxiety prevalence to be 20.3%, as quantified by GAD-7 > 10 [30]. In the present study, we found that 31.1% of students have moderate and severe anxiety (GAD-7 > 10), which represents an increase from the aforementioned study. This study’s results have important implications in serving as one time point of anxiety prevalence during the pandemic and may be of value for future investigations of COVID-19’s psychological impacts on medical students. 

Overall, we found that, during the COVID-19 pandemic, there is a high prevalence of anxiety and stress in students. We also determined that students with preexisting mental health conditions are more likely to have higher anxiety and stress levels. Furthermore, we delineated specific COVID-19 related stressors that are unique to medical students in different levels of training. 

### 4.1. Predictors of Anxiety and Stress 

The study results show that students who had higher changes in anxiety levels since March 2020 had correspondingly high levels of anxiety and stress. Even though the present study did not include baseline knowledge of stress or anxiety in the study population, the results indicate that students note that their anxiety is elevated compared to before the start of the pandemic. The impacts of COVID-19 on daily life was also found to be a predictor of anxiety and stress. COVID-19 has had significant impacts on daily life, ranging from Stay at Home orders at the time of the survey to social distancing and mask wearing practices as areas have lifted restrictions [31]. These overarching public health measures may have contributed to feelings of distress. Additionally, social isolation because of physical distancing may contribute to feelings of loneliness that may have led to increased levels of anxiety and stress. 

Furthermore, results show that self-reported changes in anxiety level since March 2020 was significantly different for students in different education levels. Third year students had higher changes in anxiety levels since March 2020 as compared to first and second year students. This data is as expected given that at the time of the survey, in addition to changes in curriculum, third year students had just recently transitioned into the clinical learning environment when the COVID-19 cases continued to rise in the United States. As the usual transition from preclinical to clinical years is normally stressful for third year students, it is imperative that special attention is needed as students transition from the didactic to clinical curriculums, especially during the pandemic or future events that may be disruptive to learning [5,9,32]. 

Predictors of stress included the student’s year in school and the rapid transition to remote learning. These factors likely impact students’ academic performance and student wellbeing and thus are important topics for medical school administrators to recognize and address. Third and fourth year students were unsure about their ability to progress through their education in a time when students were no longer allowed in or were limited in choices of clinical learning environments. Students likely feared potential exposure if they did continue their clinical rotations. For first and second year students, the transition to remote learning contributed to feelings of stress. Students found themselves having to work in their more distracting home environments, losing the social support of their peers. Both faculty and students had to adapt to the use of new technologies quickly [2]. Therefore, stressors unique to both preclinical and clinical learning environments must be quickly identified and addressed to ensure student success. 

Furthermore, study results also show that students who reported higher anxiety and stress levels compared to their peers as well as students who reported higher change in anxiety and stress levels since March 2020 is directly correlated with higher PSS-4 and GAD-7 scores. This is significant given that first, students who self-report higher normal anxiety and stress levels have heightened levels during the pandemic. Secondly, students may be self-aware of increased anxiety and stress levels based on the strong correlations of self-reported scores with PSS-4 and GAD-7 scores. However, this brings up the concern that while students may be self-aware, are the students cognizant of the extensiveness the pandemic has altered their mental health? Therefore, it is of utmost importance that mental health impacts be continuously assessed throughout the pandemic to identify students who reach concerning levels of stress and anxiety that may require clinical intervention. 

### 4.2. Unique Stressors to Each Class 

The present study also identified unique stressors for first to fourth year students. First and second year students rated higher stress due to trust in government institutions and stress due to others’ social distancing practices. These stressors are both uniquely reflective of the United States pandemic response. At the time the survey was administered, rhetoric from the national leadership frequently downplayed the severity of the virus while encouraging disregard for various individual and public safety guidelines recommended by the Centers for Disease Control and World Health Organization (WHO) [33]. As a potential result of this influence, adherence to social distancing was not a norm for much of the country [34]. First and second students’ anxiety may be stemmed from a fear of the disease spreading uncontrolled given that mask wearing in the United States has been a controversial issue and is frequently politicized [35]. There is a conflict for students between having an understanding of public health and the behaviors being modeled by community members, which may be a source of heightened anxiety. Further research is needed to know whether this effect is more pronounced in areas with a higher proportion of people who do not wear masks and do not adhere to recommended guidelines. 

For third and fourth years, the study results indicate significantly higher stress potentially due to high-stakes checkpoints in medical students’ academic career—disruption of standardized board exams that determine eligibility for residency and accessing rotations and residency. The highest stressor for third year students and second highest stressor for fourth year students was the delay and availability of standardized exams. At the time of the survey, testing centers were closed due to elevated infection rates across the country, and many students had their standardized board exams rescheduled multiple times [36]. Studying for board exams without definitive test dates may contribute to feelings of burnout for these students. The highest stressor for fourth year students was the impact of the pandemic on rotations and residency. With clinical rotations cancelled or postponed and away rotations unavailable due to safety concerns, students may have had elevated levels of stress being unable to experience or distinguish themselves to residency programs they hope to apply to as well as losing valuable in-person clinical training time [12]. Exploring options for additional enriching virtual clinical learning may potentially alleviate some concerns for upperclassmen who were unable to receive the clinical education normally offered both at home and away institutions. 

### 4.3. The Effect of Preexisting Mental Health Conditions on Anxiety and Stress Levels during the COVID-19 Pandemic 

This study reports that students with preexisting mental health conditions had significantly higher anxiety and stress levels, as well as a significantly higher percentage of reported stress attributed to COVID-19. These results are expected given that people with preexisting mental health conditions may have limited access to essential treatment and services or may have to adjust methods of receiving care, such as the switch to virtual sessions, during the pandemic. The WHO found that across 130 countries, over 60% reported disruption to mental health services for vulnerable people [37]. The higher percentage of stress attributed to COVID-19 is also expected given that people with mental health disorders have increased risk of infection and have worse outcomes than of those without [38]. Therefore, given limited or disrupted access to necessary care, compounded with being a high risk group of contracting COVID-19, these may be explanations for why preexisting mental health conditions may lead to higher stress and anxiety levels.

### 4.4. Study Limitations and Future Research 

There are several limitations to the present study, including the limited collection of demographic data. In the survey, gender and age were not collected out of privacy purposes. As the survey collected information on school name, year in school, and race, collecting gender and age may allow for easy identification of students. Several factors were taken into consideration regarding the decision to omit gender in the survey. Previous literature showed mixed results about whether gender influences medical students’ health. Hardeman et al. reported that there were no significant gender differences with respect to self-rated health in medical students [39]. Similarly, their group also did not find a significant interaction effect for depressive symptoms using an unadjusted bivariate model. Dyrbye et al. reported that there were no significant association observed between gender and serious thoughts of dropping out [40]. Age was also omitted from the survey given that a majority of students fall under the 20-25 years old category and would provide more harm than value to the study [41]. Out of privacy concerns, the survey sought to limit in collecting sensitive information as much as possible. Therefore, caution should be taken when interpreting the data given the limited demographic information.

Another important area of research that was not further developed in this study was the impact of racial disparities in stress and anxiety in medical students. Reflective of medical school enrollment demographics, the present study had a limited response in students of color. A study reaching a wider population of medical students is needed to understand how race impacted mental health in medical students during the pandemic. Black communities share a higher burden of COVID-19 infections and death due to structural disparities [42]. Asian-American communities have experienced a wave of increased stigmatization [43]. Understanding how these disparities impact mental health in medical students is important to promote access to mental health services and resilience.

Another limitation of the present study is that geographic differences in stress and anxiety in medical students during the COVID-19 pandemic were not evaluated. While institutional data was collected, the geographic region of the students’ medical school may not be used as a proxy for their physical location because many students traveled from their respective campuses when in person classes and clinical experiences were cancelled. Thus, medical school geographic regions may not accurately show geographic differences in stress and anxiety. This is an important area of further study to understand how the intensity of lockdowns and cultural differences may impact medical student stress and anxiety in different regions of the United States.

As there was not any recent, preexisting data to compare with the present study’s sample, the survey asked students to recall their own perceptions of how stress and anxiety compared to their peers before the COVID-19 pandemic. Students were provided with the definition of anxiety from the American Psychological Association prior to recalling their anxiety level to ensure students’ understanding of anxiety [44]. Measure of students’ perception of their own anxiety compared to their peers may be a proxy to understanding students’ normal anxiety levels. To that end, caution should still be taken when interpreting the data given that recalled data may not be accurate. Future studies may collect data at different time points to compare changes in stress and anxiety during events disruptive to learning.

Another limitation of the present study is that the survey did not seek to delineate the specific types of preexisting mental health conditions as reported by students. Students were only asked to denote yes or no for preexisting mental health conditions. The goal of the survey question is to provide results of whether students with preexisting mental health conditions should be prioritized and provide information for current medical students. Especially for incoming medical students with mental health conditions, it is crucial that they recognize starting school during the pandemic will be more challenging, and they may experience higher risks of stress and anxiety compared to their peers. Future studies may consider whether specific types of mental health illnesses have different influences on medical students’ stress and anxiety levels during events disruptive to learning.

Given that the data was collected between June to August of 2020, this is a time period of transition into the next level of training for many students, which may include heightened stress due to the transition. Therefore, caution should be taken when interpreting the data. However, to account for this, the present data highlight several important findings that indicate increased stress and anxiety levels may be partially attributed to the disruptions caused by pandemic. The present study shows that students had much higher rate of anxiety (31.1%) compared to preexisting data of 20.3% [30]. Students also rated novel COVID-19 stressors that have contributing to feelings of stress, and the results showed that students at different levels of training have unique stressors, which were new challenges that students were facing during the pandemic that may increase their stress and anxiety levels. Additionally, the study investigated whether year in school had an effect on students’ rating of percentage of stress and anxiety that is caused by COVID-19. First year students had the lowest rated percentage of stress and anxiety compared to other school years. However, there was large variabilities in both percentage of stress and anxiety within each class. While these differences are not significant, the trends are consistent with those seen in other stress and anxiety measures. Future studies may investigate whether different time points of training within one academic year influence students’ stress and anxiety levels, and this may pinpoint the optimal times for interventions as needed.

This study was conducted towards the start of the pandemic in the United States and reflects students’ stress and anxiety during that period, which may not reflect the students’ responses at the time of this publication. Early in the pandemic, there were many unknowns about the nature of the pandemic, the lockdowns were novel, and there was often conflicting information regarding the disease process and spread. More impactfully, the healthcare system was in high burden with increased number of patients compounded by challenges in diagnosis and treatments of confirmed cases [45]. These fears and stressors unique to medical students may not hold true today. As the pandemic has progressed since the data collection, many areas have experienced “lockdown fatigue,” and certain areas of the country are no longer requiring social distancing guidelines. Therefore, the psychological impacts of COVID-19 may have changed as personal impacts changed. 

As the pandemic continues, there is an increasing need to continue to track the mental health status of all populations, including medical students. Specifically, for medical students, understanding anxiety and stress changes due to COVID-19 will allow educators to guide recommendations on the potential implementation of psychological interventions and support strategies for students identified to be in need. Psychological care and counseling should be available, normalized, and destigmatized. Further studies may focus on delineating specific protective and risk factors as well as precautionary measures that may have contributed to lower or higher stress and anxiety levels. Furthermore, as the pandemic has no definitive end in sight, it is crucial that medical school administrators continue to monitor COVID-19’s psychological impacts on students in order to identify student groups in need and develop tailored interventions to alleviate mental health concerns. As there was not any recent, preexisting data to compare with the present study’s sample, this study asked students to recall their own perceptions of how stress and anxiety compared to their peers; thus, this presents as a limitation for the present study. Future studies may collect data at different time points to compare changes in stress and anxiety. Additionally, as the current research only encompasses 0.755% of the entire U.S. undergraduate medical student population, more research with larger sample sizes is needed to better understand the psychological impacts of COVID-19 on students nationwide. Future research may also focus on increasing participation rates in individual medical schools so that the data is more representative of their student body. Data collected now will not only benefit current students during the ongoing COVID-19 pandemic but also serve as the basis for preparations for best paths forward during future pandemics or events disrupting medical education.

## 5. Conclusions

Medical school is a high-pressure environment, and students are not immune to the added stressors presented by a global pandemic. Previous studies have shown that medical students are a population vulnerable to higher rates of mental health issues including stress and anxiety. This study shows that medical students are experiencing significant impacts due to the stress added by the pandemic and have high levels of anxiety at survey time. Results indicate a disproportionate impact on second, third, and fourth year students and those with pre-existing mental health conditions. Students in these groups may benefit from targeted intervention. Additionally, we identified COVID-19-related stressors that were unique to each class, and interventions may be implemented to address these concerns. By understanding the factors that contributed the most to stress due to COVID-19, educators may be able to formulate tailored interventions. These results may also help guide recommendations to improve the wellbeing of medical students by recognizing and addressing COVID-19’s continuous psychological impacts on different classes as well as preparing for future pandemics and other disruptive events to undergraduate medical education. As psychological interventions are more effective when intervening early, there is an urgent need for medical schools to address likely increases in stress and anxiety levels while the COVID-19 pandemic remains ongoing.

## Figures and Tables

**Table 1:** Sample Characteristics.

Characteristics	852 (100%)
Year in School	
First Year	210 (24.65)
Second Year	286 (33.57)
Third Year	180 (21.13)
Fourth Year	176 (20.66)
Race	
Black/African American	42 (4.93)
American Indian or Alaska Native	1 (0.12)
Asian	119 (13.97)
Hispanic, Latino, or of Spanish Origin	40 (4.69)
Native Hawaiian or Other Pacific Islander	1 (0.12)
White	576 (67.61)
Multiracial	60 (7.04)
Others	13 (1.53)

Values are expressed as number of students (percentage).

**Table 2:** Anxiety Levels Based on GAD-7 Scores.

Anxiety Level	First Year	Second Year	Third Year	Fourth Year	Overall
Normal	70 (33.33)	93 (32.52)	55 (30.56)	71 (40.34)	289 (33.92)
Mild	90 (42.86)	101 (35.31)	55 (30.56)	52 (29.55)	298 (34.98)
Moderate	31 (14.76)	57 (19.93)	43 (23.89)	33 (18.75)	164 (19.25)
Severe	19 (9.05)	35 (12.24)	27 (15.00)	20 (11.36)	101 (11.85)

Values are expressed as number of students (percentage).

**Table 3:** Academic, Social, and Health Stressors Related to COVID-19.

Stressors	First Year	Second Year	Third Year	Fourth Year	Overall
Academic					
Quality of Clinical Educational Experiences	5.2 ± 3.2	5.7 ± 3.0	6.3 ± 2.7	6.1 ± 2.6	5.8 ± 2.9
Remote Learning	5.8 ± 2.9	6.0 ± 2.7	5.4 ± 2.7	5.1 ± 2.7	5.6 ± 2.8
Delay and Availability of Standardized Exams	2.8 ± 3.1	3.6 ± 3.5	**7.4 ± 3.2**	7.2 ± 2.9	4.9 ± 3.8
Impact on Rotations and Residency	2.3 ± 3.1	2.5 ± 3.3	5.0 ± 3.6	**7.8 ± 2.9**	4.1 ± 3.9
Health					
Becoming Ill	6.2 ± 2.7	6.4 ± 2.5	6.5 ± 2.6	6.3 ± 2.6	6.3 ± 2.6
Death and Disability	3.6 ± 3.0	3.9 ± 3.1	4.1 ± 2.9	3.8 ± 3.0	3.9 ± 3.0
Personal Protective Equipment Availability	4.3 ± 2.7	4.4 ± 2.8	4.9 ± 2.6	4.9 ± 2.9	4.6 ± 2.7
Social					
Impact on Daily Life	6.2 ± 2.3	6.2 ± 2.4	6.6 ± 2.3	6.2 ± 2.4	6.3 ± 2.4
Social Distancing Practices of Others	6.3 ± 2.5	6.5 ± 2.6	6.2 ± 2.5	5.9 ± 2.9	6.3 ± 2.6
Trust in Government Institutions	**6.7 ± 2.6**	**7.0 ± 2.7**	6.8 ± 2.8	6.9 ± 2.7	**6.8 ± 2.7**

Values are expressed as mean score ± standard deviation. On a scale of 1 (not stressful)–10 (extremely stressful), students rated how the following stressors have contributed to feelings of stress during the COVID-19 pandemic. Bolded are the highest rated stressors for each class.

**Table 4:** Effects of Preexisting Mental Health Conditions with Higher Stress and Anxiety Levels During the COVID-19 Pandemic.

Variable	Mental Health Diagnosis	Number of Students	Mean	Standard Deviation	*p*-Value
PSS-4 Scores	Yes	227	8.08	3.00	<0.001
	No	599	6.86	2.99	
	Prefer Not to Say	26	8.88	2.88	
GAD-7 Scores	Yes	227	9.29	5.32	<0.001
	No	599	6.72	5.07	
	Prefer Not to Say	26	8.65	5.32	
Percentage of Stress Due to COVID-19	Yes	227	54.33%	21.45%	<0.05
	No	599	49.35%	24.23%	
	Prefer Not to Say	26	53.15%	21.03%	

*p*-values are as calculated using Kruskal Wallis.

**Table 5:** Effects of Perceptions of Stress and Anxiety. N = 852. *p* < 0.01 for all Spearman’s Rho values listed.

Variable	Stress Compared to Other Students (before COVID)	Anxiety Compared to Other Students (before COVID)	Changes in Anxiety Levels Since March 2020	Changes in Stress Levels Since March 2020	PSS-4	GAD-7
Stress Compared to Other Students (Before COVID)	-	0.715	0.405	0.327	0.492	0.572
Anxiety Compared to Other Students (Before COVID)	0.715	-	0.351	0.415	0.496	0.532
Changes in Stress Levels Since March 2020	0.405	0.351	-	0.751	0.551	0.570
Changes in Anxiety Levels Since March 2020	0.327	0.415	0.751	-	0.553	0.516
PSS-4	0.492	0.496	0.551	0.553	-	0.715
GAD-7	0.572	0.532	0.570	0.516	0.715	-

*p* < 0.01 for all Spearman’s correlation coefficients (Rho values) listed which show significant relationships between variables.

## Data Availability

Data available on request due to restrictions. The data presented in this study are available on request from the corresponding author. The data are not publicly available due to the sensitive nature of the data collected from students.
